# Giant anterior scleral staphyloma caused by blunt ocular trauma: a case report

**DOI:** 10.1186/s12886-023-03188-6

**Published:** 2023-11-14

**Authors:** JinBo Chen, Yang Wang, LongBin Yu, Weina Ren, Yan Sheng

**Affiliations:** 1 Ningbo Eye Hospital, Wenzhou Medical University, Ningbo, 315000 China; 2 Department of Ophthalmology, First Affiliated Hospital, College of Medicine, Zhejiang University, Hangzhou, 310003 Zhejiang China; 3 Clinical Research Center, First Affiliated Hospital, College of Medicine, Zhejiang University, Hangzhou, 310003 Zhejiang China; 4 Department of Ophthalmology, Fuyang First People’s Hospital, Hangzhou, 310003 Zhejiang China; 5 Department of Ophthalmology, The Affiliated People’s Hospital of Ningbo University, The Eye Hospital of Wenzhou Medical University (Ningbo Branch), Ningbo, 315040 China

**Keywords:** Anterior scleral staphyloma, Scleral laceration, Blunt ocular trauma, Scleral patch graft, Case report

## Abstract

**Background:**

Anterior scleral staphyloma is a relatively rare disease characterized by thinning and expansion of sclera. We described the clinical presentation, diagnosis and treatment of a case with giant anterior scleral staphyloma caused by blunt ocular trauma.

**Case presentation:**

A 24-years-old male, presented with a black cyst-like mass protruding from the right eyeball for 9 years after a history of glass crush contusion. The ultrasound biomicroscopy examination showed two cysts in the right eyeball. The larger one was about 5.92 mm*4.69 mm in size and the scleral lacerations were connected to the posterior chamber below the cyst. For treatment, resection of the anterior scleral staphyloma and the scleral patch graft transplantation was performed. The vision of the patient was improved compared with that before surgery. There were no obvious complications.

**Conclusion:**

The clinical presentation, diagnosis, and treatment of the case with giant anterior scleral staphyloma can provide a reference for the management of anterior scleral staphyloma. Surgical resection and scleral patch graft should be a good option for the treatment of giant anterior scleral staphyloma.

## Background

As an important part of the outer wall of the eyeball, the sclera supports and protects the internal eye structures, enduring the complex loads of eye movement and fluid pressure [1]. If the thickness and structure of the sclera undergo alterations due to various factors, such as ectasia, it can lead to a localized defect in the eye wall, resulting in the protrusion of internal uveal tissue called a staphyloma [2]. The reasons for sclera thinning include congenital abnormalities and pathological damages under increasing or normal intraocular pressure (IOP). Compared with posterior scleral staphyloma often caused by high myopia [3], anterior scleral staphyloma is a relatively rare disease. The common causes of anterior scleral staphyloma include trauma, surgery, inflammation, glaucoma, high myopia, malnutrition, and dysplasia. Clinically, anterior scleral staphyloma commonly occurs near the perilimbal sclera, and is often complicated with other diseases, such as keratoconus [4] and glaucoma. To the best of our knowledge, due to the special form of traumatic mechanism, there are few reports on this kind of scleral staphyloma and a systematic clinical description is lacking. Herein, we reported a case with giant anterior scleral staphyloma caused by blunt ocular trauma. The clinical presentation, diagnosis and treatment of this case were described and discussed.

## Case presentation

**Fig. 1:**
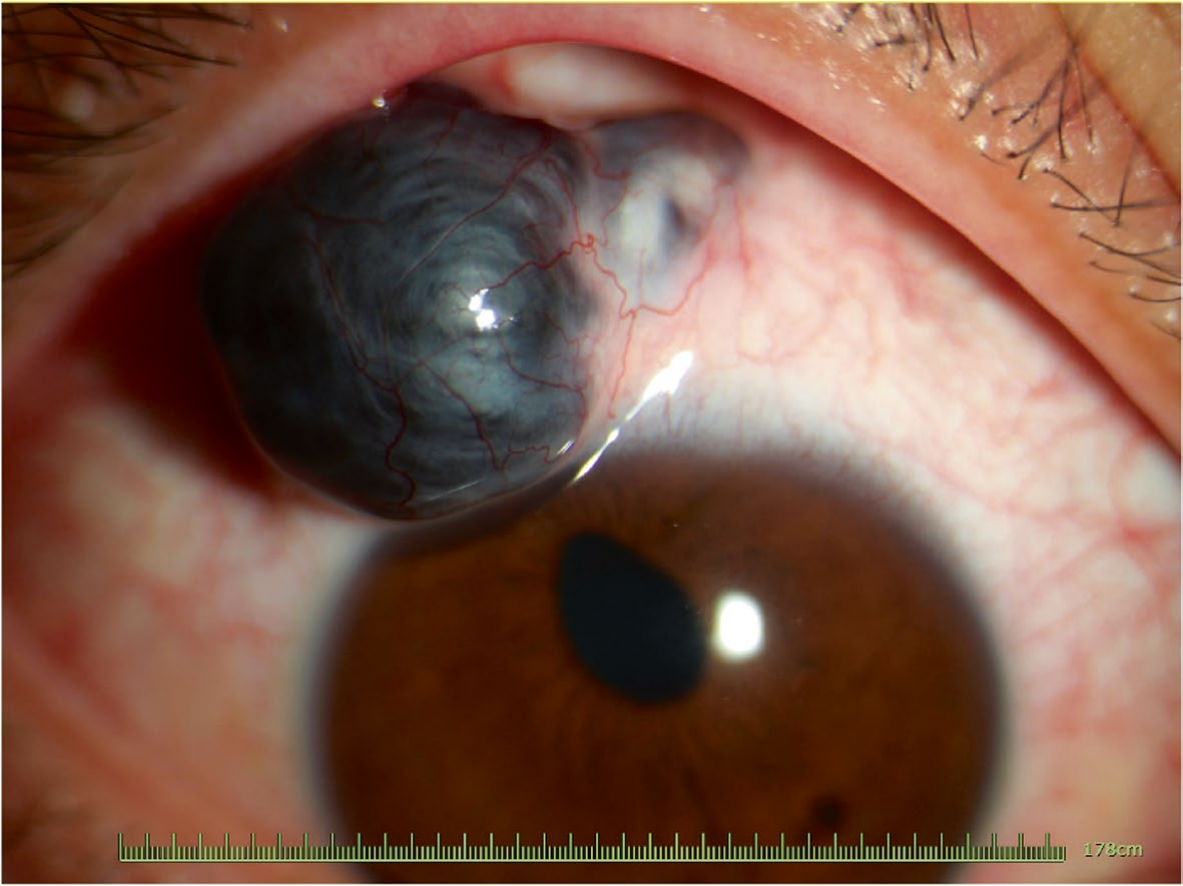
Intraocular examination of the patient: The black cyst and an irregular pupil

**Fig. 2:**
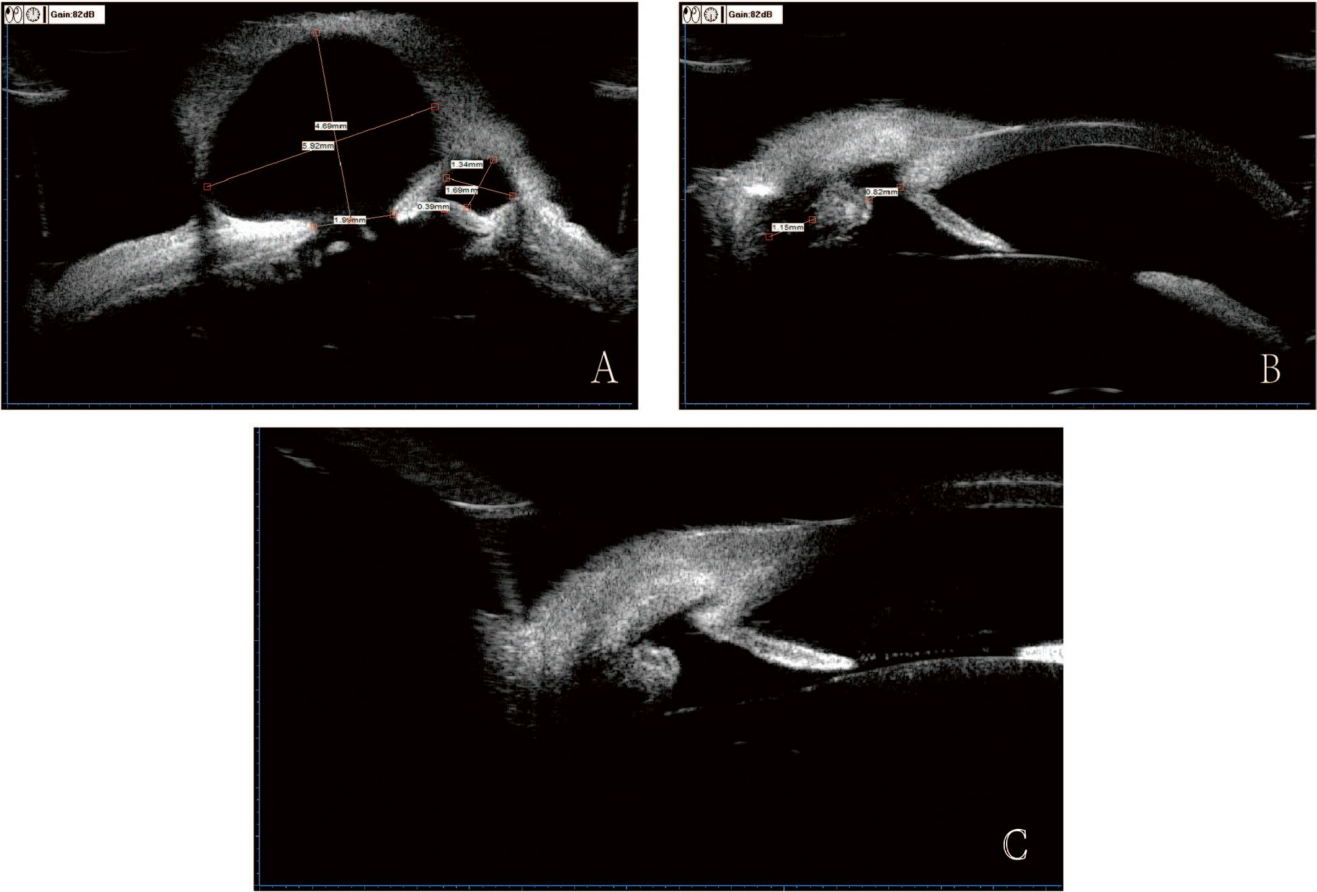
UBM examination of the patient. **a** Preoperative image showed that two scleral lacerations were connected to the posterior chamber and the cyst. The maximum size of cyst was 5.92 mm*4.69 mm. **b** Postoperative image at one month after surgery showed that the lacerations were covered with the sclera graft and were repaired. **c** Postoperative image at three months after surgery showed that the scleral graft fits the lacerations better

A 24-year-old male was admitted with a progressively enlarged black grape-shaped cyst protruding from the upper temporal region of the right eyeball and foreign body sensation for 9 years. He had a glass crush contusion in the right eye 9 years ago. Additionally, the glass panel slid towards his head during the process of handling, resulting in a laceration and blunt contusion to the upper eyelid of the right eye. The ophthalmic examination revealed that the Best-Corrected Visual Acuity (BCVA) measured 20/25 in the right eye and 20/20 in the left eye. The IOP was 20.6 mmHg in the right eye and 14.0 mmHg in the left eye. There was a strip of scar tissue about 10 mm in length at 12 o’clock on the right upper eyelid. Anterior segment examination revealed a giant staphyloma, which was approximately 7 mm in diameter and located at the 10–12 o’clock of the corneoscleral limbus. The staphyloma had a vague and thin layer of fibrous tissue, and was covered by a bulbar conjunctiva. The rest of the sclera and cornea were normal without obvious traces of previous injuries. Intraocular examination (Fig. 1) showed that the pupil was not round and was eccentric toward the staphyloma. A clear lens was observed without obvious signs of traumatic cataracts. Fundus examination of the posterior segment revealed no retinal anomalies or retinal detachment. The ultrasound biomicroscopy (UBM) examination showed that the left eye was completely normal, but there were two cysts in the right eye. The larger cyst was about 5.92 mm*4.69 mm in size, with the scleral laceration (about 1.9 mm and 0.39 mm) connecting to the posterior chamber below the cyst (Fig. 2a). Based on the above findings, the patient was diagnosed with anterior scleral staphyloma preoperatively. Considerating the patient’s history of progressive enlargement of the staphyloma and the psychological distress caused by its visible protrusion, particularly when he looks downward, surgical intervention has been recommended.

**Fig. 3:**
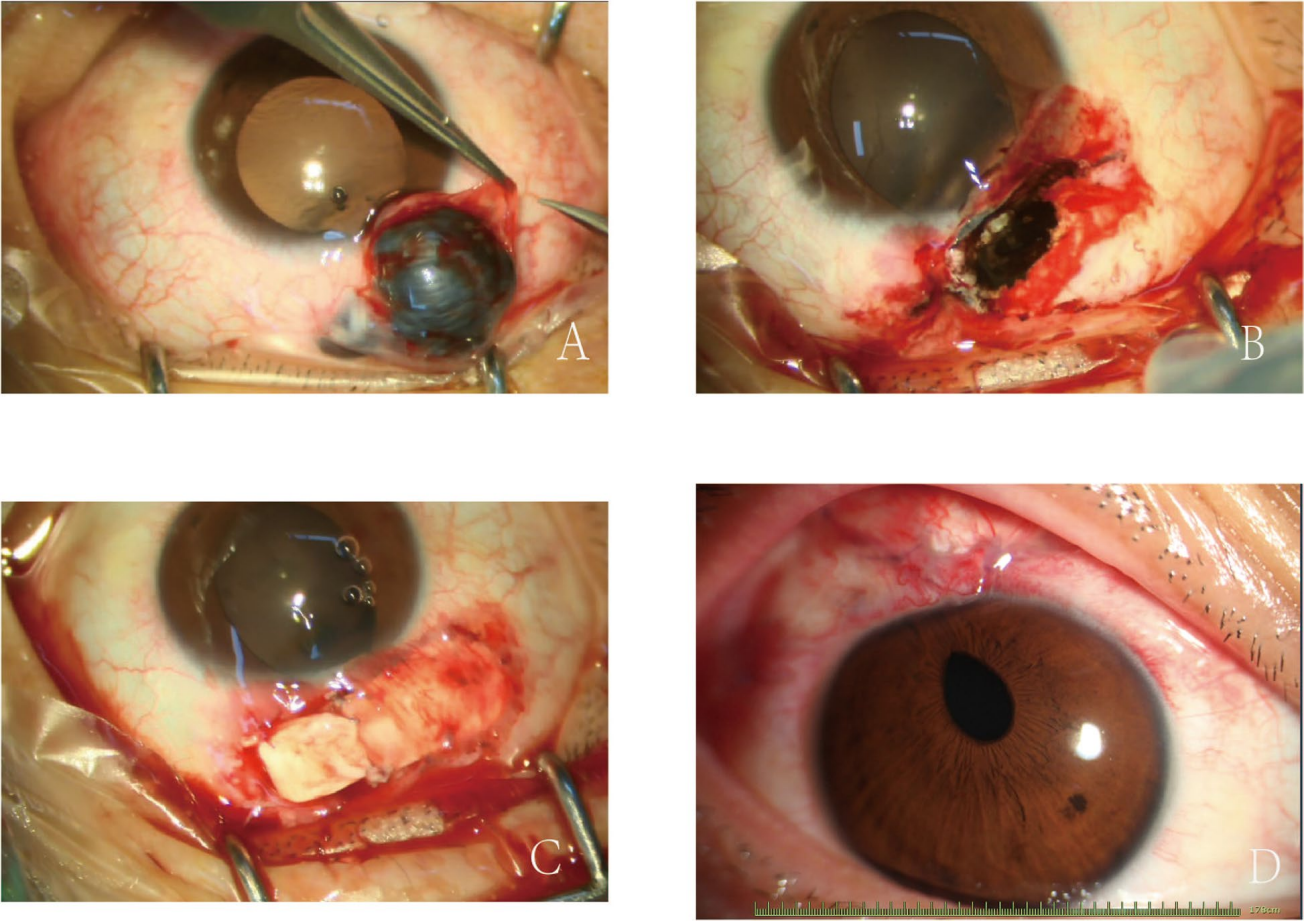
Intraoperative and postoperative findings. **a** The staphyloma was exposed after separating the conjunctiva on the surface. **b** When the staphyloma tissue was removed, the scleral laceration below can be observed. **c** The sclera graft was sutured on the laceration. **d** Postoperative image of the anterior segment

On August 16, 2022, the patient received resection of the anterior scleral staphyloma and allograft scleral graft transplantation under retrobulbar anesthesia. The staphyloma was injected with viscoelastic material to maintain IOP before its resection (Fig. 3a). The resected cystic tissue was pathologically examined. After resection of the staphyloma, the scleral laceration was observed (Fig. 3b), which was then covered with the scleral patch that had been well rinsed with 0.1% amikacin-prepared saline (Fig. 3c). Postoperatively, tobramycin dexamethasone drops and levofloxacin drops were given four times a day for anti-inflammatory and anti-infective treatment. IOP was monitored three times a day during hospitalization.

**Fig. 4:**
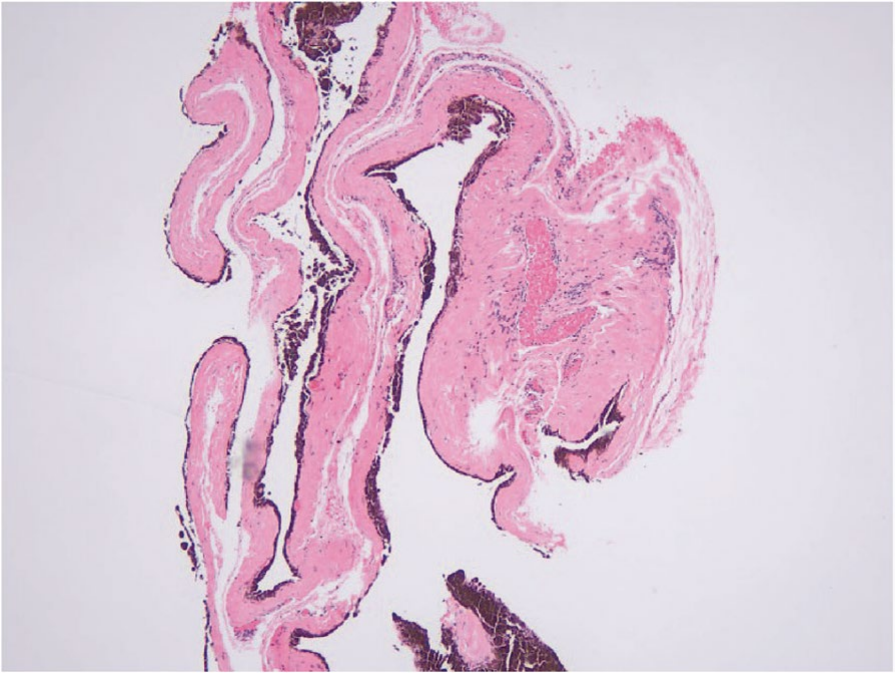
Pathological image of the staphyloma tissue

One week after the surgery, the BCVA of the right eye was 20/20, and the IOP was 8 mmHg. Postoperative pathological examination of the staphyloma showed fibrous tissues and blood vessels. Pigment cells were attached to the inner layer and there was no obvious epithelial tissue (Fig. 4), which confirmed the diagnosis of scleral staphyloma. Postoperatively, the anterior segment examination showed the anterior chamber, the pupil, and the iris were consistent with those before (Fig. 3d). One month after surgery, the IOP of the right eye was 12.3 mmHg. The UBM examination showed that the sclera graft covered the laceration, which was obviously smaller than before (Fig. 2b). The other clinical findings were consistent to those at one week after surgery. Three months after surgery, the IOP of the right eye was 14.5 mmHg. The UBM examination showed that the sclera graft fits the lacerations better than that before, and no obvious graft thinning was observed (Fig. 2c).

## Discussion and conclusion

We described a rare case with giant anterior scleral staphyloma caused by blunt ocular trauma. In other reports [2, 5], the anterior scleral staphyloma is often complicated with damages to the cornea, ciliary body, lens, vitreous or retina. However, the case in this report had anterior scleral staphyloma and scleral laceration but was not complicated with other ocular structural damage. The size of the staphyloma was larger compared with that of previously reported cases [5–7]. To the best of our knowledge, this study is the first to report detailed information on a giant anterior scleral staphyloma case caused by trauma. Therefore, this case may be valuable for evaluating the development of anterior scleral staphyloma. Additionally, good postoperative recovery in this case may also provide a reference for the treatment of anterior scleral staphyloma.

The sclera makes up about 85% of the outer layer of the eye wall. Meanwhile, it is a connective tissue composed mainly of elastic and collagen fiber. The thickness of the scleral varies with its anatomical position, ranging from ~ 0.5 mm at the equator to ~ 0.8 mm at the perilimbal sclera [1]. The ciliary muscle and iris are attached to the inner surface of the anterior sclera. In response to external stretching or blunt trauma, the curl of the sclera fiber is gradually straightened, and the scleral stiffness gradually increases [8]. When the force of external stretching or blunt trauma exceeds the elastic limit of the fiber, the sclera tends to break and form a scleral laceration.

In this patient, the most significant history was a previous injury by glass contusion, and scars from the blunt contusion were visible on the upper eyelid. We consider that the patient may have suffered a blunt contusion to the right upper eyelid, and that the sclera at 10–12 o’clock below the injured eyelid may also be injured. The medial scleral tissue was partially ruptured due to overstretching, but some scleral elastic fibers were retained on the inside. Therefore, the injury did not cause a full scleral laceration. With the disappearance of external pressure, the damaged area of the sclera becomes the most vulnerable. The aqueous humor and nearby iris root may bulge from this vulnerable area, resulting in pupil deformation and the formation of black hemispherical cysts. Fortunately, there was no other intraocular complication in this patient.

The preliminary diagnosis of the anterior scleral staphyloma is not difficult, but it still needs to be differentiated from ciliary leiomyoma and choroidal melanoma. Their appearance can be very similar. A large number of spindle smooth muscle cells can be observed in the tissue section of ciliary leiomyoma, and specific actin can be observed by immunohistochemistry [9]. Because of a large number of pigmented cells, choroidal melanoma can be preliminarily identified by a light transmission test. Therefore, the final affirmative diagnosis should be based on the pathological results, the medical history and the results of auxiliary examinations.

Progressive scleral staphyloma and scleral thinning have become indications for scleral reinforcement surgery [2, 10–12]. The development of staphyloma is often related to the location and size of the scleral laceration, IOP, and the tenacity. The staphyloma size in this patient gradually increased during the past 9 years, reaching diameter of approximately 7 mm, but the IOP remained at the upper limit of normal. In addition, the staphyloma is located between the ciliary body and the limbus cornea, where the sclera is weakened by the presence of the anterior ciliary veins and Schlemm’s canal [11, 12]. The patient was also still working as a porter with a high probability of reoccurrence of trauma. All these reasons increase the likelihood of further progression of the staphyloma and even perforation, so prophylactic scleral staphyloma surgery is essential.

However, before choosing a surgical approach, the patient must be thoroughly evaluated. The choice of approach depends on the location and size of the staphyloma, the condition of the conjunctiva, the age of the patient, previous surgery or injury, visual acuity, expected vision after the intervention, and the opportunity for follow-up treatment [5]. The larger staphyloma require more complex methods, e.g., patch grafts, such as scleral patch graft [2, 6, 13], tibial periosteal patch graft [2], corneoscleral grafts, fascia lata [14], pericardial patches [15], aotric tissue [16] and tragal perichondrium [7] (Table 1). The prognosis of most cases is satisfactory, except for the graft dissolution and IOP increase in a few cases [2]. Although a variety of materials have been used to deal with the repair of scleral disorders, the donor sclera has putative advantages due to the ready availability of scleral tissue and its ability to adequately provide a large area of donor material. Scleral tissue also provides a natural curvature of the desired site, which is a definite advantage for anterior scleral staphylomas and reduces operative source aberrations [10]. In addition, the donor scleral tissue appears to be well tolerated with little inflammatory response [2, 17]. Effective conjunctiva and amniotic membrane to cover the scleral graft can greatly improve the survival rate of scleral transplantation.

**Table 1:** The summary of surgical results of scleral diseases

Author	Disease Type (Number of cases)	Surgical Graft	Number of Successful Cases (Total Number)	Reasons For Failure/Complications (Number of cases)
Rajagopal R [2]	Anterior Staphyloma	Scleral Patch Graft	10(15)	IOP Rise (4); Graft Melt (1)
		Tibial Periosteal Patch Graft	1(2)	Recurrent Staphyloma
Mor JM [9]	Ciliary Leiomyoma	Scleral Patch Graft	1(1)	——
Sangwan VS [13]	Necrotizing Scleritis (7) Scleral Fistula (1) Ciliary Staphyloma (1) Scleral Rupture (3) Scleral Thinning (1)	Scleral Patch Graft	7(13)	Endophthalmitis (1) Cataract (1) Graft Dehiscence (1) Necrotization (1) Persistent Hypotony (1) Thinning (1)
Barman M [6]	Uveal Melanoma (5) Scleromalacia (2) Uveoscleral Nevi (2) Scleral Necrosis (1)	Scleral Patch Graft	8(10)	Graft Retraction (1) Graft Thinning (1)
Ozcan AA [5]	Scleral Defects	Corneoscleral Grafts	3(3)	——
		Scleral Patch Grafts	3(4)	Recurrence Defect
		Fascia Lata	1(1)	——
Kobtan H [14]	Surgically Induced Scleral Necrosis	Fascia Lata	2(2)	——
Weissgold DJ [15]	Scleral Buckles Exposure	Pericardial Patches	3(4)	Graft Melt
Merz EH [16]	Bilateral Scleromalacia Perforans	Aortic Tissue	1(1)	**——**
Chun YS [7]	Ahmed Glaucoma Valve Tube Exposure	Tragal Perichondrium	1(1)	——

Therefore, we used the allogeneic scleral graft to repair the scleral laceration in this case. Follow-up showed that BCVA of the right eye at 1 week after surgery was 20/20, which is improved postoperatively, and the IOP at 1 month after surgery was 12.3 mmHg. The patient’s condition was relatively stable, and he was satisfied with the results of the operation. The effective coverage of conjunctival flap can provide nutritional support for scleral graft and reduce immune response [5]. Additionally, Hwan and Kim reported on the efficiency of amniotic membrane transplantation (AMT) used in conjunction with scleral grafts, especially in cases of adjacent conjunctival defects. Rapid re-epithelialization of the conjunctiva and marked improvement in visual acuity was noted in most patients [18]. Furthermore, three of the seven observed scleral surgical management patients had improved visual acuity to varied degrees [5]. Therefore, scleral transplantation combined with conjunctival cover in this patient can effectively change the ocular surface microenvironment and improve the visual quality of patients. In general, comprehensive risk factor evaluation, management of ocular comorbidities, and timely, meticulous surgery can improve prognosis.

We discussed the clinical presentation, diagnosis, and treatment of a case with giant anterior scleral staphyloma caused by blunt ocular trauma. Our case may provide a reference for the management of anterior scleral staphyloma. To inhibit the progression of giant staphyloma, we still recommend early detection and treatment for anterior scleral staphyloma. Additionally, we are able to perform surgery using a variety of techniques, although a scleral patch graft is preferred.

## Data Availability

The datasets used and analysed during the current study available from the corresponding author on reasonable request.

## Abbreviations

IOP: Intraocular pressure
BCVA: Best corrected visual acuity
UBM: The ultrasound biomicroscopy
AMT: Amniotic membrane transplantation
